# Short-term outcomes of totally robotic versus robotic-assisted distal gastrectomy for gastric cancer: a single-center retrospective study

**DOI:** 10.1186/s12957-024-03484-5

**Published:** 2024-09-04

**Authors:** Shan-Ping Ye, Can Wu, Rui-Xiang Zou, Dong-Ning Liu, Hong-Xin Yu, Jin-Yuan Duan, Tai-Yuan Li

**Affiliations:** Department of General Surgery, The First Affiliated Hospital, Jiangxi Medical College, Nanchang University, No. 17 Yongwaizheng Street, Nanchang, Jiangxi Province 330006 China

**Keywords:** Gastric cancer, Robotic distal gastrectomy, Short-time outcomes

## Abstract

**Background:**

Totally robotic distal gastrectomy (TRDG) is being used more and more in gastric cancer (GC) patients. The study aims to evaluate the short-term efficacy of TRDG and robotic-assisted distal gastrectomy (RADG) in the treatment of GC.

**Methods:**

We retrospectively collected the clinical data of patients who underwent TRDG or RADG, of which 60 patients were included in the study: 30 cases of totally robotic and 30 cases of robotic-assisted. The short-term efficacy of the two groups was compared.

**Results:**

There was no significant difference in the clinicopathological data between the two groups. Compared to RADG, TRDG had less intraoperative blood loss(*P* = 0.019), less postoperative abdominal drainage(*P* = 0.031), shorter time of exhaust( *P* = 0.001) and liquid diet(*P* = 0.001), shorter length of incision(*P*<0.01), shorter postoperative hospital stays(*P* = 0.033), lower postoperative C-reactive protein(CRP)(*P* = 0.024) and lower postoperative Visual Analogue Scale(VAS) scores(*P* = 0.048). However, no significant statistical differences were found in terms of total operation time(*P* = 0.108), number of lymph nodes retrieved(*P* = 0.307), time for anastomosis(*P* = 0.450), proximal resection margin(*P* = 0.210), distal resection margin(*P* = 0.202), postoperative complication(*P* = 0.506), total hospital cost(*P* = 0.286) and postoperative white blood cell(WBC)(*P* = 0.113).

**Conclusions:**

In terms of security and technology, TRDG could serve as a better treatment method for GC.

## Introduction

Gastric cancer (GC) is the fifth most common cancer worldwide and the fifth leading cause of cancer death[1]. With the development of technology and the reduction of risk factors, its incidence is gradually decreasing^2^. Since the first report of laparoscopic gastrectomy for the treatment of GC in 1994[3], the minimally invasive surgery had been widely accepted. (laparoscopic assisted distal gastrectomy) LADG has better short-term and long-term outcomes compared to open distal gastrectomy[4, 5]. And compared to LADG, there was also study [6] that has confirmed that total laparoscopic distal gastrectomy (TLDG) is equally safe and feasible. However, LADG has some technical limitations, such as unavoidable physiological tremor, limited mobility and two-dimensional visualization[7], which make it difficult to perform precise lymph node dissection, thus affecting the prognosis.

To overcome the limitations of laparoscopic surgery, The robot has emerged. Compared to laparoscopic surgery, robot has better 3D vision, easier instrument manipulation and the elimination of physiological tremor[8], which are more conducive to precise lymph node dissection and separation of complex anatomical structures. At the same time, the early results of robot-assisted gastric cancer treatment are satisfactory [9, 10].

However, the safety and feasibility of totally robotic distal gastrectomy (TRDG) is not fully clear. Therefore, we designed this study to compare the safety and advantages and disadvantages of the two surgical methods.

## Method

### Study population and data collection

In this retrospective cohort study, we retrospectively collected and analyzed the clinical and pathological data of patients who underwent distal gastrectomy at the First Affiliated Hospital of Nanchang University from January 2023 to May 2024. Distal gastrectomy was performed on 64 gastric cancer patients, including 31 totally robotic cases and 33 robotic cases. 4 patients were excluded due to liver metastasis, pelvic metastasis, pancreatic metastasis, preoperative chemotherapy, and combined resection of other organs. Finally, 60 patients met the criteria, with 30 totally robotic cases and 30 robotic cases. The study was approved by the hospital’s Institutional Review Board and complied with the Declaration of Helsinki. All patients signed informed consent forms before the surgery.

Inclusion criteria: ^1^ age: 18–80 years, ^2^ no distant metastasis, ^3^ signed informed consent, ^4^ preoperative examination confirmed that the tumor was located in the middle and lower part of the stomach.

Exclusion criteria: ^1^ conversion to open surgery, ^2^ combined multi-organ resection, ^3^ preoperative neoadjuvant therapy, ^4^ emergency surgery,^5^ incomplete clinical data.

All patients underwent preoperative esophagogastroduodenoscopy, biopsy, and enhanced chest and abdominal computed tomography for diagnosis, staging, and evaluation. Tumor staging was based on the criteria from the 7th edition of the American Joint Committee on Cancer (AJCC) guidelines. Postoperative pain was evaluated by the standard clinical visual analog scale (VAS) of 0–10, with 0 representing no pain and 10 representing the worst pain imaginable.

### Surgical technique

The surgical method was chosen by the patients and their family after full understanding the potential advantages and disadvantages of TRDG and RADG, and they signed the informed consent form. According to the Japanese Gastric Cancer Association guidelines [^11^, all patients underwent standard curative distal gastrectomy and D2 lymph node dissection, performed by an experienced team. Most surgical procedures for RADG and TRDG are the same, including anesthesia and positioning, trocar placement, abdominal exploration, placement of robotic surgical systems, and lymph node dissection. The above processes can refer to previous study ^12^. The following are different surgical procedures, which are generally divided into three stages for TRDG. ^1^ Specimen resection: Extend a 60 mm linear stapler from the auxiliary port, resect the specimen at a position of no less than 5 cm above the tumor, and then remove about 70% of the distal stomach, along with the greater omentum and surrounding adipose lymphoid tissue. The assistant places the resected specimen into a specimen bag, then tightens the suture of the bag and places it on the lower abdomen. ^2^ Gastrointestinal anastomosis: Lift the jejunum at about 20 centimeters from the Treiz ligament, and then use enterectomy about 1 centimeter in size from the jejunal wall. The same method is used for gastrotomy about 1 centimeter from the residual stomach. Finally, use the 45 mm linear cutting closure through the auxiliary port and insert it between the stomach and jejunum. Finally, perform lateral anastomosis of the gastrointestinal tract, and suture the remaining openings with 3.0 barbed thread. The residual end of the duodenum is reinforced with continuous suture and buried into the suture. After abdominal lavage, there was no significant bleeding, and a drainage tube was placed through the robot arm hole 2. ^3^ Specimen removal: Remove the specimen approximately 3 centimeters from the observation port. Finally, suture the entire abdominal wall layer by layer. For RADG, following the completion of the same steps, the robotic surgical system is removed. We made a midline incision in the upper abdomen and placed a protective ring, followed by specimen resection and Billroth II anastomosis. The duodenal stump is reinforced. If no significant bleeding is observed after abdominal irrigation, a drainage tube is inserted, and the abdomen is closed layer by layer.

### Parameters for observation and evaluation

The patient’s general demographic data includes age, gender, body mass index, and ASA classification. The patient’s pathological data includes tumor location, differentiation type, tumor diameter, number of harvested lymph nodes, number of metastatic lymph nodes, perineural invasion, lymphovascular invasion, and TNM stage. The patient’s surgical data includes operation time and intraoperative blood loss. Postoperative inflammatory response data includes white blood cell count and C-reactive protein.

### Date analysis

All statistical analyses were performed using SPSS 26.0. All data were first tested for normality, and normally distributed data were expressed as mean ± standard deviation, while non-normally distributed data were expressed as median and range, using independent sample t-test or Mann-Whitney U test. Categorical data were analyzed using chi-square test or Fisher’s exact test, and presented as frequency and percentage. P-value less than 0.05 was considered statistically significant.

## Results

### Clinical baseline

The study compared Gender, Age, Body Mass Index (BMI), C-reactive Protein(CRP), White Blood Cell(WBC), Hemoglobin(HB), Carcino Embryonic Antigen(CEA), tumor diameter, Tumor Node Metastasis stage(TNM stage), and American Society of Aneshesiologists (ASA), and there was no significant statistical difference between the two groups in the Table 1.

**Table 1:** Comparison of baseline data between totally robotic group and robotic group

Variable	Total robot	Robot	*P* value
Gender, n, (%)			0.095
Male	24(40%)	17(28.33%)	
Female	6(10%)	13(21.67%)	
Age, years	59(8.84)	60(8.66)	0.608
BMI, Kg/m2	22.66(2.65)	22.41(3.66)	0.762
Diameter of neoplasm, cm	3.52(1.32)	4.07(1.61)	0.157
TNM stage, n(%)			0.310
I	10(16.67%)	8(13.33%)	
II	10(16.67%)	6(10%)	
III	10(16.67%)	16(26.67%)	
Preoperative C-reactive protein , mg/L	1.38(0.1-20.14)	1.9(0.06–44.14)	0.198
Hemoglobin, g/L	116 ^22^	113 ^16^	0.617
Preoperative white blood cell, count/L	4.49(0.28–8.59)	5.28(0.22–9.01)	0.169
CEA, ng/ml	3.25(0.54–11.67)	2.94(0.31–21.03)	0.464
ASA, n(%)			0.671
II	4(6.67%)	2(3.33%)	
III	26(43.33%)	28(46.67%)	

### Short-term outcomes

The short-term results of the two groups of patients are shown in the Tables 2, 3. In terms of intraoperative blood loss, the totally robotic group was significantly less than the robotic-assisted group (100(50–200) ml vs. 115(50–400) ml, *P* = 0.019), and the postoperative volume of abdominal drainage in the totally robotic group was also lower than that in the robotic-assisted group (195(95–300) ml vs.215(85–310) ml, *P* = 0.031). In terms of postoperative rehabilitation, the TRDG group has shorter time of exhaust (23.5(20–30) vs. 26(22–36) h, *P* = 0.001) and liquid diet (38.5(35–45) vs. 41(37–51) h, *P* = 0.001) and shorter postoperative hospital stays (8.5 [7–12] vs. 9 [7–14] day, *P* = 0.033). As for the postoperative wound aesthetics, the TRDG group has shorter length of incision(3 [2–6] vs. 6 [5–9] cm, *P*<0.01).Regarding postoperative C-reactive protein, the level was lower in the TRDG group (*P* = 0.024). In terms of postoperative quality of life, the VAS score in the totally robotic group was significantly lower than that in the robotic-assisted group(*P* = 0.048). However, no significant statistical differences were found in terms of total operation time(*P* = 0.108), number of lymph nodes retrieved(*P* = 0.307), time for anastomosis(*P* = 0.450), proximal resection margin(*P* = 0.210), distal resection margin(*P* = 0.202), total hospital cost(*P* = 0.286), and postoperative white blood cell(WBC)(*P* = 0.113). In terms of postoperative complications, there was no statistical difference between the two groups(*P* = 0.506), with 4 cases of complications in the totally robotic group, including 3 cases of bowel obstruction and 1 case of pneumonia, and 7 cases of complications in the robotic-assisted group, including 5 cases of bowel obstruction and 2 cases of pneumonia.

**Table 2:** Comparison of perioperative indexes between totally robotic group and robotic group

Variable1	Total robot	Robot	*P* value
Total operative time, min	240(170–290)	220(185–300)	0.108
Time for anastomosis, min	70(58–78)	71.5(58–80)	0.45
Estimated blood loss, ml	100(50–200)	115(50–400)	0.019
Time to exhaust, h	23.5(20–30)	26(22–36)	0.001
Time to liquid diet, h	38.5(35–45)	41(37–51)	0.001
Length of incision, cm	3 [2–6]	6 [5–9]	0.000
Proximal resection margin, cm	6 [5–8]	6 [5–9]	0.210
Distal resection margin, cm	6 [5–8]	6 [5–9]	0.202
Postoperative volume of abdominal drainage, ml	195(95–300)	215(85–310)	0.031
Postoperative hospital stays, day	8.5 [7–12]	9 [7–14]	0.033
Harvested lymph nodes	21(13–49)	24(10–51)	0.307
Perineural invasion, n (%)			0.192
+	10(16.67%)	16(26.67%)	
-	20(33.33%)	14(23.33%)	
Vascular invasion, n (%)			0.301
+	13(21.67)	18(30%)	
-	17(28.33%)	12(20%)	
Total hospitalization cost, $	9357	6834	0.286
Postoperative complication, n(%)	4(6.67%)	7(11.67%)	0.506
Bowel obstruction	3(5%)	5(8.33%)	
Gastroparesis	0	0	
Anastomotic leakage	0	0	
Pneumonia	1(1.67%)	2(3.33%)	
Complication of Clavien-Dindo classifcation ≥ 3, n	0	0	

**Table 3:** Comparison of postoperative C-reactive protein, white blood cell and VAS scores between totally robotic group and robotic group

	Total robot	Robot	*P* value
Postoperative C-reactive protein, mg/L			0.024
Day 1	30.79(28.41)	45.03(26.60)	
Day 3	58.33(45.08)	95.59(58.18)	
Day 5	37.43(37.95)	44.43(26.06)	
VAS scores			0.048
Day 1	2.63(0.96)	3(1.26)	
Day 3	1.43(0.89)	2.57(0.77)	
Day 5	0.83(0.79)	1.43(0.72)	
Postoperative white blood cell, count/L			0.113
Day 1	9.17(3.17)	10.59(3.43)	
Day 3	7.54(3.11)	8.00(1.93)	
Day 5	7.66(2.78)	7.14(2.06)	

Day 1 first day after surgery, Day 3 third day after surgery, Day 5 fifth day after surgery

## Disscusion

Now, LADG has become a mature and safe feasible technology for the treatment of gastric cancer[13, 14, 15]. As technology advances, the daVinci surgical system has increasingly gained recognition, however its full potential remains unclear. In clinical practice, we observed that certain patients in the TRDG group exhibited improved short-term postoperative outcomes, leading to the conduct of this study. Intraoperative bleeding volume is an important indicator for evaluating the quality of surgery, and in the study, we found that the intraoperative bleeding volume of TRDG was less than that of RADG, which is consistent with the results of previous studies[16, 17]. This can be attributed to the robotic surgical system, as the 3D vision of the robot can help the surgeon identify more delicate structures, and the more flexible joint movements[18]can reduce vascular damage. At the same time, the robotic surgical system filters out physiological tremors, and the grip is stronger, thereby reducing vascular damage caused by tremors or mirror retraction.

The recovery of gastrointestinal function is crucial for the postoperative recovery of patients, and the indicators reflecting the recovery of gastrointestinal function include the time of flatus and the time of liquid or semi-liquid diet intake. In our study, the exhaust time and feeding fluid time of TRDG were significantly lower than those of RADG. This may be due to the strong stimulation of the gastrointestinal tract by external dry air during the open abdominal resection and anastomosis of RADG specimens, while excessive traction affects gastrointestinal function. Pain is an important indicator reflecting the quality of life of patients after surgery. In this study, the TRDG group had a lower pain score than the RADG group and had statistical significance. The reduction in postoperative pain can be attributed to the fact that robotic surgical systems have less trauma compared to open surgery, making it easier for the surgeon to separate tissues and reduce damage to the gastrointestinal mesentery, thereby reducing exudation and stimulation [19, 20]. The important indicator of postoperative wound aesthetics is the incision length. We found that the incision length in the TRDG group was significantly lower than that in the RADG group, which is of great significance for the postoperative psychological recovery of patients. Currently, there are few studies comparing the intraperitoneal drainage volume between the TRDG group and the RADG group. However, this study found that compared to the RADG group, the TRDG group had less drainage volume, which is also due to the robot surgical system [21] and complete minimally invasive techniques. In addition, patients undergone TRDG had a shorter hospital stay than that in RADG group; this could be explained with the same causes mentioned above.

The surgical time in the TRDG group was longer than that in the RADG group, but there was no significant difference. For oncological outcomes, the number of lymph node dissection, proximal and distal resection margins were similar in both groups. In terms of surgical safety, there was no significant difference in the incidence of postoperative complications between the two groups.

Postoperative inflammatory response is an important indicator for assessing surgical quality and postoperative recovery[22]. More surgical trauma normally can lead to higher levels of inflammation [23]. We use white blood cells and CRP to evaluate postoperative inflammatory response. In our study, the CRP levels in the TRDG group were significantly lower than those in the RADG group, similar to previous studies [24], while there was no significant difference in white blood cell levels. A study [25] have shown that inflammation is one of the factors promoting tumor occurrence and metastasis, so a lower validation level in the TRDG group may be beneficial for patient prognosis.

Finally, our research also has some limitations. As this is a retrospective study, selection bias is inevitable, and due to the limitations of single center studies and surgical approaches, our sample size is small. And the long-term outcomes are lack. Therefore, more randomized controlled studies with larger sample sizes are needed for further research.

## Conclusion

In summary, TRDG is a safe and feasible treatment method, with better short-term outcomes compared to RADG, such as less intraoperative blood loss, reduced abdominal drainage, and lower postoperative pain scores. With the improvement of the technical level of surgeons, TRDG will become the standard surgical procedure for treating distal gastric cancer.

## Data Availability

No datasets were generated or analysed during the current study.

## Abbreviations

ASA: American Society of Aneshesiologists
BMI: Body Mass Index
CEA: Carcino Embryonic Antigen
CRP: C-Reactive Protein
C-D: Clavien-Dindo
GC: Gastric Cancer
LADG: Laparoscopic-Assisted Distal Gastrectomy
HB: Hemoglobin
RADG: Robot-Assisted Distal Gastrectomy
SD: Standard Deviation
TNM: Tumor Node Metastasis
TRDG: Totally Robotic Distal Gastrectomy
TLDG: Totally Laparoscopic Distal Gastrectomy
VAS: Visual Analogue Scale
WBC: White Blood Cell
